# Patterns, motivations, and determinants of dietary supplement use among physically active adults in Eastern Saudi Arabia: a cross-sectional survey

**DOI:** 10.3389/fpubh.2026.1734477

**Published:** 2026-01-22

**Authors:** Majed M. Alhumaid, Maryam A. Alobaid, Mohamed A. Said

**Affiliations:** 1 Department of Physical Education, College of Education, King Faisal University, Al-Ahsa, Saudi Arabia; 2 Higher Institute of Sports and Physical Education of Kef, University of Jendouba, Jendouba, Tunisia

**Keywords:** anthropometric characteristics, cross-sectional survey, dietary supplements, motivation, physically active adults, prevalence, training frequency

## Abstract

**Background:**

Dietary supplement use is widespread among physically active adults; however, the determinants of this behavior remain insufficiently understood, particularly within the Saudi context. This study aimed to examine the prevalence of dietary supplement use and its associations with physical activity, demographic, and behavioral characteristics among adults in the Eastern Province of Saudi Arabia.

**Methods:**

A cross-sectional survey was conducted among 755 physically active adults. Data were collected on physical characteristics (weight, height, and BMI), demographic characteristics (age, gender, marital status, employment, income, and education), and behavioral characteristics (training frequency and motivation). Differences between supplement users and non-users were assessed using independent-samples *t*-tests, two-way ANOVA with *post hoc* Tukey’s tests, and chi-square tests. Effect sizes were reported as Cohen’s d, η^2^, and Cramer’s V, respectively.

**Results:**

Among the 755 participants, 505 (66.9%) reported using dietary supplements, including 250 males (65.96%) and 255 females (67.82%). No significant differences were observed between users and non-users in age, weight, height, or BMI (*t* = 0.32–1.68, *p* = 0.093–0.975, *d* = 0.02–0.19). ANOVA confirmed sex-related differences in height and weight (*η*^2^ = 0.20–0.47) and minor differences in BMI (*η*^2^ = 0.02), with female supplement users being younger and male users having slightly higher BMIs. Supplement use was significantly associated with body type (*V* = 0.12), occupation (*V* = 0.41), and education (*V* = 0.21), but not income. Male supplement users exercised more than four times per week (64.4%), significantly higher than female users (42.9%) (*χ*^2^ = 71.18, *p* < 0.001, *V* = 0.31), whereas training motivations did not differ across sex × supplement use groups (*χ*^2^ = 20.49, *p* = 0.154, *V* = 0.16).

**Conclusion:**

Dietary supplement use is common among physically active adults in the Eastern Province of Saudi Arabia and is largely unaffected by basic anthropometric or demographic factors. However, it is shaped by sex-specific exercise patterns and educational and occupational contexts, highlighting the need for targeted public health efforts to promote safe, informed supplement use through improved regulation and education.

## Background

Adequate nutrition is essential for meeting the physiological demands of regular physical activity (PA), regardless of whether the goal is to enhance performance, increase muscle mass, manage weight, or improve overall health (1). However, widespread misconceptions and limited nutritional knowledge have led many individuals to adopt unbalanced diets high in fats and calories but lacking essential proteins, vitamins, and minerals (2). Over time, such nutritional imbalances have contributed to the growing prevalence of degenerative and metabolic disorders. Consequently, dietary supplements have emerged as a perceived solution to compensate for nutritional deficiencies and support physical and mental well-being, including maintaining energy, reducing fatigue, and minimizing injury risk (1, 3, 4). Key nutrients such as B vitamins, iron, magnesium, and protein contribute to energy metabolism, cognitive function, and recovery (5–7). Despite their popularity, many users self-prescribe without professional guidance, highlighting the need for informed and safe supplementation practices.

The United States’ Dietary Supplement Health and Education Act of 1994 defines dietary supplements as products intended to supplement the diet by providing nutrients such as vitamins, minerals, herbs, amino acids, or other dietary substances (8). They are commonly available in tablets, capsules, powder, or liquid form and are promoted for maintaining health, enhancing performance, and preventing disease. Popular examples include multivitamins, minerals, calcium, and omega-3 fatty acids. Despite their widespread use, most consumers self-prescribe without professional supervision, raising safety concerns related to misuse and overconsumption (9). Notably, research evidence on their benefits remains inconsistent, particularly among individuals who already maintain balanced diets (10).

Globally, the use of dietary supplements continues to rise, with prevalence rates ranging from 69 to 94% among elite athletes and similarly high levels among recreational exercisers (1, 11). Marketing campaigns frequently emphasize the necessity of supplements for success, often downplaying the critical role of balanced nutrition, systematic training, and adequate recovery (6). In the Arab world, research on supplement use remains limited. Reported prevalence rates include 70% among gym members in Jordan (12), 39% among physically active adults in the United Arab Emirates (13), and 36.3% among individuals exercising in gyms in Beirut, Lebanon (14). In Saudi Arabia, previous studies conducted primarily in Riyadh and other central cities have reported prevalence rates ranging from 22% to 53% (15–17).

Saudi Arabia’s Vision 2030 initiative has spurred major social and economic reforms that promote PA and health awareness. The expansion of fitness centers, the inclusion of women in organized sports, and the hosting of international sporting events have cultivated a growing culture of exercise and well-being (18). These developments have also fueled rapid growth in the dietary supplement industry. Saudi Arabia represents the largest dietary supplement market in the Middle East, with a market value projected to reach US$366.94 million by 2026, and an expected annual growth rate of 8.04% through 2026 (19). Purchasing supplements is relatively easy, as they are sold over the counter in pharmacies, private stores, gyms, and online marketplaces. Many individuals who engage in physical and athletic activities often consume supplements without fully considering their potential side effects, primarily because of the widespread belief that they are state-regulated and inherently safe performance enhancers (20).

However, most research in Saudi Arabia has focused on elite athletes or populations in central regions, leaving other areas, such as the Eastern Province, underrepresented. Nonetheless, known for its industrial diversity, multicultural population, and evolving fitness culture, the Eastern Province provides a unique context for examining dietary supplement use behaviors. Therefore, this study aimed to determine the prevalence of dietary supplement use in the Eastern Province and to identify its associated demographic, anthropometric, and behavioral characteristics. This study posited the following hypotheses:

*H1*: Dietary supplement use will vary significantly by demographic characteristics.

*H2*: Users and non-users will differ significantly in physical characteristics.

*H3*: Dietary supplement use will correlate with variations in PA patterns and motivations.

This study is part of a larger research project on dietary supplement use among physically active individuals in the Eastern Province of Saudi Arabia. Part I of this project (this study) focuses on usage patterns and associated factors, establishing a foundational profile for subsequent analysis. Part II will build upon these findings to examine perceived advantages, negative consequences, and potential doping-related risks using multivariate predictive models.

## Methods

### Study design and population

This descriptive cross-sectional study was conducted over 3 months (November 2023 to January 2024) and included physically active adults residing in the Eastern Province of Saudi Arabia. The study did not aim to compare governorates or specific forms of physical activity; rather, it sought to provide an overall picture of dietary supplement use among active adults in the region. The research adopted a quantitative design and relied on closed-ended questionnaire items.

The study sample consisted of Saudi adults living in the Eastern Province who consistently participated in physical exercise (e.g., fitness club members, gym participants, and independent exercisers). The inclusion criteria were: (a) Saudi nationality, (b) age 18–60 years, (c) consistent PA for ≥12 consecutive weeks, (d) provision of informed consent, and (e) submission of a complete questionnaire. Individuals with chronic diseases limiting PA were excluded.

### Sampling and data collection

A non-probability quota sampling strategy was used due to the absence of a comprehensive sampling frame for physically active adults in the Eastern Province of Saudi Arabia and the practical constraints associated with recruiting this population using probability-based methods. Because gym members and recreational exercisers are not registered in any unified database from which individuals could be randomly selected, probability sampling was not feasible and was therefore ruled out. Quota sampling enabled balanced representation across key sociodemographic groups (sex and age categories: 18–29, 30–44, and ≥45 years) while maintaining operational feasibility in terms of time, cost, and access to recruitment sites. This approach enhanced subgroup coverage and supported the study’s exploratory objective of examining patterns and associations; however, it does not permit full probabilistic generalization to the wider provincial population.

Participants were recruited using a multi-channel strategy that comprised direct collaboration with fitness clubs, online distribution via social media platforms (X, Instagram, and WhatsApp), and on-site recruitment at supplement stores and community events. To increase diversity and eliminate selection bias, data were collected from different cities in the Eastern Province, including Dammam, Khobar, Qatif, and Al-Ahsa. The limitations associated with this sampling method are acknowledged, and the findings should be interpreted with consideration of potential restrictions in external validity beyond the province.

The required sample size was estimated using Cochran’s formula, considering a 95% confidence level, a 5% margin of error, and a 22% predicted prevalence based on previous research (15). This calculation yielded a minimum required sample size of 264, which was increased to 330 to account for anticipated non-responses and dropouts.

Data collection occurred in two phases. First, a pilot study with 34 participants assessed the clarity, structure, and comprehensibility of the questionnaire, resulting in minor linguistic revisions. In the second phase, data were collected from the remaining participants. The study was approved by the Ethics Committee of King Faisal University’s Deanship of Scientific Research (Ref: KFU-REC-2023-OCT-ETHICS1589) and conducted in accordance with the Declaration of Helsinki. All participants provided written or electronic informed consent, and confidentiality was ensured through anonymization and secure, encrypted data storage.

### Research instrument

#### Design overview

The data were collected using a structured questionnaire originally developed by Ionescu et al. (21) to investigate the patterns, motivations, and drivers of dietary supplement use, which was translated into Arabic and adapted to the Saudi context by our research team. First, the original English text was independently translated into Arabic by two bilingual experts. Next, the two translations were amalgamated to produce a single Arabic version, which was then back-translated into English by a native speaker unfamiliar with the original document. Then, the back-translated version was juxtaposed with the original English version to verify conceptual and semantic coherence.

The questionnaire consisted of two major parts. Part I comprised 8 items that collected data on demographic and socioeconomic variables: sex, age, weight, height, marital status, personal education level, occupation, and household income. Part II comprised 14 items that collected data on different aspects of dietary supplement use, which were organized into four thematic domains: PA Patterns (Items 1–3), which investigated exercise frequency, PA type, and key motivations for PA; Supplement Consumption Behavior (Items 4–8), which assessed dietary supplement use status (user/non-user), consumption goals, perceived advantages, frequency of intake, and self-reported physiological changes after supplementation; Side Effects and Supplement Types (Items 9 and 10), which documented adverse effects and supplement types used; and Determinants of Supplement Choice (Items 11–14), which examined product selection reasons, purchasing sources, label-reading behavior, and propensity to promote supplements to others.

#### Technical specifications

Body mass index (BMI) was calculated from self-reported height and weight using the conventional formula: weight (kg) / height-squared (m^2^) and classified according to the World Health Organization’s criteria: underweight (<18.5 kg/m^2^), normal weight (18.5–24.9 kg/m^2^), overweight (25.0–29.9 kg/m^2^), and obese (≥30.0 kg/m^2^).

The questionnaire consisted primarily of multiple-choice questions, some of which allowed multiple selections. Open-ended “Other” responses were used only when a participant’s answer did not appear among the listed options, allowing them to introduce an alternative response rather than to provide narrative elaboration. The measurement scales were as follows. Consumption frequency was measured on a 5-point scale (1 = *monthly*, 2 = *weekly*, 3 = *over four times monthly*, 4 = *over four times weekly*, and 5 = *often*). Incentives for supplement consumption were measured on a 5-point nominal scale (1 = *other*, 2 = *aesthetic incentive*, 3 = *enjoyment of physical exercise*, 4 = *medical motivation*, and 5 = *several reasons*). Usage objectives were measured on a 5-point scale (1 = *decrease body fat*, 2 = *enhance muscle mass*, 3 = *reduce weight*, 4 = *augment muscle strength*, 5 = *post-exercise recovery or multiple objectives*). Perceived benefit was measured on a 5-point cognitive scale (1 = *no benefit*, 2 = *enhanced energy*, 3 = *weight reduction*, 4 = *muscular hypertrophy*, and 5 = *supplementation for nutrients not readily acquired from diet*). Self-efficacy for metabolic enhancement was measured on a four-point scale (1 = *no change*, 2 = *improvement >10%*, 3 = *improvement >20%*, and 4 = *improvement >30%*).

Categorical variables, including supplement sources and product selection motivations, were numerically encoded to enable sophisticated statistical analyses. Items 5–14 pertain to the behavioral, motivational, and outcome-related dimensions of dietary supplement usage. In accordance with the project structure outlined in the Introduction, these items are included as part of the instrument description but are not subjected to analytical interpretation in the present manuscript. Their comprehensive analysis—including perceptions of benefits, adverse effects, factors influencing product selection, and the recommendation item (Item 14)—is allocated to Part II, where these aspects will be examined through multivariate and predictive modeling.

#### Validation and reliability

The Arabic version of the questionnaire underwent a comprehensive psychometric evaluation to ensure its validity and reliability. It was assessed for face, content, and construct validity as follows. The face validity of the questionnaire was examined by a panel of five experts in PA and sports nutrition using a 4-point relevance scale. All items were rated appropriately, indicating high face validity.

The content validity of the questionnaire was assessed by the same panel of experts, with item relevance rated on a 4-point scale. The item-level and scale-level content validity indices were both 1.0, indicating perfect expert agreement and extensive construct coverage.

The construct validity of the questionnaire was evaluated by assessing both convergent and discriminant validity using a pilot sample of 34 participants (17 men and 17 women) from the study population. Convergent validity was examined by correlating the questionnaire with the Dietary Supplement Questionnaire (22). Pearson’s correlation coefficients exceeded 0.50 across most domains, indicating strong convergence between the two measures (Supplementary Table S1). Discriminant validity was assessed by comparing the questionnaire with the Arabic version of the Physical Activity Scale for Individuals with Physical Disabilities (23). All Pearson’s correlation coefficients were below 0.30 (Supplementary Table S2), confirming that the questionnaire measures a construct distinct from PA level.

The participants in the pilot study were also asked to provide feedback on item clarity, and minor linguistic refinements were made accordingly. The revised questionnaire achieved full response completion when re-administered 10 days later, indicating strong comprehension and stability over time.

The reliability of the questionnaire was examined using the test–retest approach, which demonstrated high temporal stability across all subscales. The overall correlation coefficient reached 0.89, while all subscale values exceeded 0.70 (Supplementary Table S3), indicating satisfactory consistency and reproducibility of responses.

Collectively, these findings confirm that the Arabic version of the questionnaire exhibits robust construct validity and robust reliability, supporting its use as a reliable tool for assessing dietary supplement consumption and related behaviors among physically active individuals.

### Statistical analysis

All statistical analyses were performed using SPSS Statistics (version 26; IBM Corp., Armonk, NY, USA), with a two-tailed *p* < 0.05 considered statistically significant. The data was screened and cleaned before analysis. Normality was assessed using standard criteria for skewness (|skewness| < 2) and kurtosis (|kurtosis| < 7), supplemented by visual examination of Q–Q plots. Univariate outliers were defined as values exhibiting an absolute *Z*-score exceeding 3 (|*Z*| > 3). Extreme values were winsorized to reduce their influence while maintaining the integrity and rank order of the data, substituting them with the nearest non-outlier value within the variable distribution. The participants’ sociodemographic characteristics, PA patterns, and supplement-related behaviors were summarized descriptively, using means ± standard deviations (SDs) for normally distributed continuous variables and frequencies (percentages) for categorical variables.

Continuous variables were compared between dietary supplement users and non-users using independent samples *t*-tests, and among sex × dietary supplement use groups using two-way analysis of variance (ANOVA) with *post-hoc* Tukey’s tests. Categorical variables were compared between dietary supplement users and non-users using chi-square (*χ*^2^) tests. To ensure transparency and interpretability, effect sizes were estimated using eta-squared (*η*^2^) for ANOVA (small ≈ 0.01, medium ≈ 0.06, and large ≈ 0.14), Cohen’s *d* for *t*-tests (small ≈ 0.2, medium ≈ 0.5, and large ≈ 0.8), and Cramer’s *V* for chi-square tests (small ≈ 0.10, medium ≈ 0.30, and large ≈ 0.50).

## Results

### Participants’ characteristics

A total of 793 questionnaires were returned, of which 38 were excluded due to incomplete responses (<90% completion), non-serious or patterned answers, or failure to meet the inclusion criterion of regular physical activity. The final sample comprised 755 participants, exceeding the planned sample size.

The sample included 379 males (50.2%) and 376 females (49.8%), reflecting balanced sex representation. Participants’ ages ranged from 18 to 50 years, with a mean of 29.61 ± 8.06 years. They had a mean weight of 71.35 ± 18.05 kg, a mean height of 166.82 ± 10.37 cm, and a mean BMI of 25.53 ± 5.60 kg/m^2^, reflecting a generally healthy population with moderate anthropometric variability (Table 1).

**Table 1:** Participants’ general characteristics.

Variable	Category	Total *n* (%)	Male *n* (%)	Female *n* (%)	*χ* ^2^	*p*	*V*
Sex	Male	379 (50.2)	—	—	—	—	—
	Female	376 (49.8)	—	—	—	—	—
Body type	Underweight	30 (4.0)	2 (0.5)	28 (7.4)	30.4	**<0.001**	0.201
	Healthy weight	384 (50.9)	185 (48.8)	199 (52.9)			
	Overweight	251 (33.2)	147 (38.8)	104 (27.7)			
	Obese	90 (11.9)	45 (11.9)	45 (12.0)			
Marital status	Not reported	15 (2.0)	13 (3.5)	2 (0.5)	39.8	**<0.001**	0.229
	Single	419 (55.5)	217 (57.2)	202 (53.8)			
	Married	291 (38.5)	121 (31.9)	170 (45.1)			
	Divorced	30 (4.0)	28 (7.4)	2 (0.5)			
Occupation	Unemployed	131 (17.4)	97 (25.5)	34 (9.2)	126.4	**<0.001**	0.409
	Government	190 (25.2)	40 (10.6)	150 (39.6)			
	Private sector	246 (32.6)	109 (28.7)	137 (36.4)			
	Student	188 (24.9)	133 (35.1)	55 (14.8)			
Monthly Household Income (SAR)	No income	154 (20.4)	100 (26.3)	54 (14.5)	22.0	**<0.001**	0.171
	<5,000	117 (15.5)	58 (15.4)	59 (15.6)			
	5,000–9,999	165 (21.9)	87 (22.9)	78 (20.8)			
	10,000–14,999	147 (19.5)	63 (16.5)	84 (22.4)			
	15,000–19,999	90 (11.9)	39 (10.4)	51 (13.5)			
	≥20,000	82 (10.9)	32 (8.5)	50 (13.2)			
Education level	Intermediate	10 (1.3)	6 (1.6)	4 (1.1)	17.06	**0.002**	0.150
	Secondary	159 (21.1)	83 (21.8)	76 (20.3)			
	Bachelor’s	389 (51.5)	211 (55.6)	178 (47.5)			
	Diploma	131 (17.4)	44 (11.7)	87 (23.0)			
	Postgraduate	66 (8.7)	35 (9.3)	31 (8.2)			

*χ*^2^, chi-square statistic; *p*, probability value; *V*, Cramér’s V (effect size); SAR, Saudi Riyal; *n*, number of participants. Bold values indicate statistical significance (*p* < 0.05).

Regarding body type, 384 participants (50.9%) had a healthy weight, 251 (33.2%) were overweight, and 90 (11.9%) were obese. BMI differed significantly by sex (*χ*^2^ = 30.4, *p* < 0.001, *V* = 0.201), with males more frequently overweight and females more frequently underweight. Most participants were single (*n* = 419, 55.5%), followed by married (*n* = 291, 38.5%) and divorced (*n* = 30, 4.0%); 15 participants (2.0%) did not disclose marital status. Regarding occupation, participants were primarily employed in the private sector (*n* = 246, 32.6%), government (*n* = 190, 25.2%), or education (*n* = 188, 24.9%). Most reported a monthly household income of 5,000–14,999 SAR (41.4%), and the majority held a bachelor’s degree (*n* = 389, 51.5%).

### Behavioral characteristics and PA patterns

The participants’ behavioral characteristics are presented in Table 2. Over half (*n* = 406, 53.8%) reported exercising more than four times per week, and 162 (21.5%) identified as an athlete. Exercise frequency was significantly higher among males than females (*χ*^2^ = 67.15, *p* < 0.001, *V* = 0.298). Most participants reported multiple motivations for PA (*n* = 399, 52.8%), followed by enjoyment (*n* = 134, 17.7%), aesthetics (*n* = 120, 15.9%), and health reasons (*n* = 82, 10.9%). Motivations did not differ significantly by sex (*χ*^2^ = 3.03, *p* = 0.696, *V* = 0.063).

**Table 2:** Participants’ behavioral characteristics.

Variable	Category	Total (*n*, %)	Males (*n*, %)	Females (*n*, %)	*χ* ^2^	*p*	*V*
Training frequency	Once a month	59 (7.8%)	6 (1.2%)	53 (14.2%)	67.152	**<0.001**	0.298
	Once a week	33 (4.4%)	16 (4.2%)	17 (4.6%)			
	>4 times/month	95 (12.6%)	31 (8.1%)	64 (17.2%)			
	>4 times/week	406 (53.8%)	246 (64.4%)	160 (42.9%)			
	Basically, an athlete	162 (21.5%)	83 (21.7%)	79 (21.2%)			
Motivation for training	Enjoyment	134 (17.7%)	67 (17.5%)	67 (18.0%)	3.028	0.696	0.063
	Aesthetic	120 (15.9%)	67 (17.5%)	53 (14.2%)			
	Medical	82 (10.9%)	36 (9.4%)	46 (12.3%)			
	Financial gain	13 (1.7%)	7 (1.8%)	6 (1.6%)			
	Social integration	7 (0.9%)	3 (0.8%)	4 (1.1%)			
	Multiple factors	399 (52.8%)	202 (52.9%)	197 (52.8%)			

*χ*^2^, chi-square statistic; *p*, probability value; *V*, Cramér’s V (effect size); *n*, number of participants. Bold values indicate statistical significance (*p* < 0.05).

### Prevalence of dietary supplement use

Among the participants, 505 (66.6%) reported taking dietary supplements, while 250 (33.4%) did not. The number of users and non-users was generally balanced and did not differ significantly between males (65.96% vs. 34.04%) and females (67.82% vs. 32.18%, *χ*^2^ = 0.294, *p* = 0.588, *V* = 0.02).

### Differences in age and anthropometric parameters by sex and dietary supplement use

The participants’ age and anthropometric parameters are presented by sex and dietary supplement use in Table 3. The distribution of age, weight, height, and BMI did not differ significantly between users and non-users (*t* = 0.32–1.68, *p* = 0.093–0.975, *d* = 0.02–0.19), suggesting that these characteristics do not strongly predict dietary supplement use. Expected sex-based differences were observed, with males taller and heavier than females (*F* = 61.93–221.66, *p* < 0.001, *η*^2^ = 0.20–0.47), while BMI differences were smaller but significant (*F* = 5.39, *p* = 0.001, *η*^2^ = 0.02).

**Table 3:** Participants’ age and anthropometric characteristics by sex and dietary supplement use.

Variable	Group	*n*	Mean	SD	Comparison	*F*/*t*	*p*	*η*^2^/*d*
Age (years)	Users	505	29.62	7.94	User vs. Non-user	0.32	0.975	0.02
	Non-users	250	29.60	8.32				
	Male: non-user	132	31.21	8.33	Sex × Supp. Use	7.91	**<0.001**	0.03
	Male: user	250	30.87	7.66				
	Female: non-user	118	27.80	7.98				
	Female: user	255	28.39	8.03				
Weight (kg)	Users	505	72.04	18.73	User vs. Non-user	1.49	0.136	0.13
	Non-users	250	69.96	16.52				
	Male: non-user	132	76.89	16.47	Sex × Supp. Use	61.93	**<0.001**	0.20
	Male: user	250	80.39	19.01				
	Female: non-user	118	62.20	12.73				
	Female: user	255	63.85	14.36				
Height (cm)	Users	505	166.77	10.21	User vs. Non-user	−0.19	0.851	0.02
	Non-users	250	166.92	10.69				
	Male: non-user	132	173.67	9.05	Sex × Supp. Use	221.66	**<0.001**	0.47
	Male: user	250	173.92	7.85				
	Female: non-user	118	159.37	6.54				
	Female: user	255	159.76	6.85				
BMI (kg/m^2^)	Users	505	25.77	5.78	User vs. Non-user	1.68	0.093	0.19
	Non-users	250	25.04	5.20				
	Male: non-user	132	25.57	5.58	Sex × Supp. Use	5.39	**0.001**	0.02
	Male: user	250	26.59	6.17				
	Female: non-user	118	24.45	4.69				
	Female: user	255	24.97	5.25				

SD, standard deviation; F/t, F-statistic (ANOVA) or *t*-statistic (independent *t*-test); *p*, probability value; η^2^, eta-squared (effect size for ANOVA); *d*, Cohen’s d (effect size for *t*-tests); BMI, body mass index. Bold values indicate statistical significance (*p* < 0.05).

*Post-hoc* Tukey’s tests showed small but significant differences in age and BMI across the sex × supplement-use groups. Female participants were younger than males among both users (28.39 ± 8.03 vs. 30.87 ± 7.66 years) and non-users (27.80 ± 7.98 vs. 31.21 ± 8.33 years; *p* = 0.003–0.005, *η*^2^ = 0.03). For BMI, male users (26.59 ± 6.17 kg/m^2^) had higher values than female users (24.97 ± 5.25 kg/m^2^) and female non-users (24.45 ± 4.69 kg/m^2^). Male non-users (25.57 ± 5.58 kg/m^2^) also had higher BMI than female users (24.97 ± 5.25 kg/m^2^, *p* = 0.003–0.006, *η*^2^ = 0.02). Overall, these results indicate that dietary supplement use is mainly independent of basic anthropometric characteristics, highlighting the likely influence of psychological, behavioral, or knowledge-based factors in this population.

Cohen’s *d* was calculated for the *t*-test comparing users vs. non-users, and *η*^2^ was calculated for the ANOVA comparing the sex × dietary supplement use groups. No significant differences were found between users and non-users in the total sample, suggesting that physical characteristics do not predict dietary supplement use. As expected, sex explains moderate variation in weight and height.

### Differences in demographic characteristics by sex and dietary supplement use

The participants’ demographic characteristics are presented by sex and dietary supplement use in Table 4. Participants with normal weight predominated across all subgroups, while those who were underweight were more common among female users (*n* = 20, 8.0%) than among male (*n* = 2, 0.8%) users and non-users (*n* = 1, 0.76%). Body type (*χ*^2^ = 33.855, *p* < 0.001, *V* = 0.12), occupation (*χ*^2^ = 127.089, *p* < 0.001, *V* = 0.41), and education level (*χ*^2^ = 34.287, *p* = 0.001, *V* = 0.21) differed significantly across sex × dietary supplement use groups, but not monthly household income (*p* = 0.067). These findings indicate that dietary supplement use is moderately associated with sex-specific patterns in body type, occupation, and education level but not with monthly household income.

**Table 4:** Participants’ demographic and anthropometric characteristics by sex and dietary supplement use.

Variable	Category	Male Non-Users (*n* = 132)	Male Users (*n* = 250)	Female Non-Users (*n* = 118)	Female Users (*n* = 255)	*χ* ^2^	*p*	*V*
Body Type	Underweight	1 (0.76%)	2 (0.8%)	9 (7.63%)	20 (8.0%)	33.855	**<0.001**	0.12
	Normal weight	71 (53.8%)	115 (46.0%)	67 (56.8%)	130 (51.0%)			
	Overweight	47 (35.6%)	93 (37.2%)	28 (23.7%)	69 (27.1%)			
	Obesity	13 (9.8%)	40 (16.0%)	14 (11.9%)	36 (14.1%)			
Occupation	Unemployed	11 (8.3%)	23 (9.2%)	30 (25.4%)	65 (25.5%)	127.089	**<0.001**	0.41
	Government	58 (43.9%)	96 (38.4%)	12 (10.2%)	30 (11.8%)			
	Private sector	45 (34.1%)	94 (37.6%)	38 (32.2%)	71 (27.8%)			
	Student	18 (13.6%)	37 (14.8%)	38 (32.2%)	89 (34.9%)			
Monthly Household Income (SAR)	No income	18 (13.6%)	36 (14.4%)	26 (22.0%)	64 (25.1%)	23.877	0.067	0.11
	<5,000	19 (14.4%)	39 (15.6%)	21 (17.8%)	41 (16.1%)			
	5,000–9,999	26 (19.7%)	59 (23.6%)	33 (28.0%)	59 (23.1%)			
	10,000–14,999	32 (24.2%)	54 (21.6%)	15 (12.7%)	42 (16.5%)			
	15,000–19,999	22 (16.7%)	31 (12.4%)	12 (10.2%)	25 (9.8%)			
	≥20,000	15 (11.4%)	31 (12.4%)	11 (9.3%)	24 (9.4%)			
Education Level	Intermediate	0 (0%)	2 (0.8%)	5 (4.2%)	4 (1.6%)	34.287	**0.001**	0.21
	Secondary	26 (19.7%)	50 (20.0%)	24 (20.3%)	59 (23.1%)			
	Bachelor’s	66 (50.0%)	118 (47.2%)	69 (58.5%)	142 (55.7%)			
	Diploma	24 (18.2%)	60 (24.0%)	10 (8.5%)	26 (10.2%)			
	Postgraduate	16 (12.1%)	20 (8.0%)	10 (8.5%)	24 (9.4%)			

*χ*^2^, chi-square statistic; *p*, probability value; *V*, Cramér’s V (effect size); SAR, Saudi Riyal; *n*, number of participants. Bold values indicate statistical significance (*p* < 0.05).

### Differences in behavioral characteristics by sex and dietary supplement use

The behavioral patterns, encompassing training frequency and motivations, were broadly similar between dietary supplement users and non-users (Table 5). Most participants engaged in frequent training, with more than half exercising more than four times per week among users (51.9%) and non-users (57.6%). Additionally, about one-fifth identified primarily as athletes among both users (22.4%) and non-users (19.6%). Just over half of participants reported multiple motivations for training among users (52.1%) and non-users (54.4%). In contrast, few participants reported financial gain or social integration as motivations (<2%).

**Table 5:** Participants’ behavioral characteristics by sex and dietary supplement use.

Variable	Category	Male Non-Users (*n* = 132)	Male Users (*n* = 250)	Female Non-Users (*n* = 118)	Female Users (*n* = 255)	*χ* ^2^	*p*	*V*
Training Frequency	Once a month	1 (0.8%)	5 (2.0%)	17 (14.4%)	36 (14.1%)	71.18	**<0.001**	0.31
	Once a week	3 (2.3%)	13 (5.2%)	6 (5.1%)	11 (4.3%)			
	>4 times/month	11 (8.3%)	20 (8.0%)	19 (16.1%)	45 (17.6%)			
	>4 times/week	92 (69.7%)	154 (61.6%)	52 (44.1%)	108 (42.4%)			
	Primarily athlete	25 (18.9%)	58 (23.2%)	24 (20.3%)	55 (21.6%)			
Motivation for Training	Enjoyment	16 (12.1%)	51 (20.4%)	28 (23.7%)	39 (15.3%)	20.49	0.154	0.16
	Aesthetic	22 (16.7%)	45 (18.0%)	15 (12.7%)	38 (14.9%)			
	Medical	18 (13.6%)	18 (7.2%)	10 (8.5%)	36 (14.1%)			
	Financial gain	1 (0.8%)	6 (2.4%)	2 (1.7%)	4 (1.6%)			
	Social integration	2 (1.5%)	1 (0.4%)	0 (0%)	4 (1.6%)			
	Multiple motives	73 (55.3%)	129 (51.6%)	63 (53.4%)	134 (52.5%)			

*χ*^2^, chi-square statistic; *p*, probability value; *V*, Cramér’s V (effect size); *n*, number of participants. Bold values indicate statistical significance (*p* < 0.05).

Training frequency differed significantly among the sex × dietary supplement use groups (*χ*^2^ = 71.18, *p* < 0.001, *V* = 0.31), with a medium effect size. Post-hoc comparisons among non-users showed that males were significantly more likely to train >4 times/week than females (*χ*^2^ = 28.47, *p* < 0.001, *V* = 0.35). In contrast, females were more likely to train once/month than males (*χ*^2^ = 18.45, *p* = 0.001, *V* = 0.25) or once/week (*χ*^2^ = 7.92, *p* = 0.005, *V* = 0.18). No significant differences were noted between those who trained >4 times/month and those who were primarily athletes.

Among users, male participants were significantly more likely to train >4 times per week than female participants (*χ*^2^ = 36.72, *p* < 0.001, *V* = 0.38), while females were more likely to train >4 times/month than males (*χ*^2^ = 10.3, *p* = 0.004, *V* = 0.21). No significant differences were observed for once/month, once/week, or primarily athlete categories (*p* > 0.05). These findings indicate consistent sex-specific patterns in training frequency, with males generally engaging in higher-frequency training, regardless of dietary supplement use, and females showing greater representation in lower-frequency training categories.

The motivation for training did not differ significantly among the sex × dietary supplement use groups (*χ*^2^ = 20.49, *p* = 0.154, *V* = 0.16). Therefore, these findings indicate that dietary supplement use is primarily independent of training motives but is partially correlated with training frequency, particularly highlighting elevated physical activity levels among males.

## Discussion

This study comprehensively analyzed the patterns, motivations, and factors associated with dietary supplement use among physically active adults in the Eastern Province of Saudi Arabia. The estimated prevalence of dietary supplement use was high (66.6%), exceeding prevalence rates reported in prior studies conducted in Riyadh (35%–53%) and neighboring Gulf countries (16, 17). This elevated prevalence likely reflects the rapid expansion of a fitness culture, improved access to gyms and wellness facilities under Saudi Vision 2030, and the growing availability of nutritional products. However, it should be highlighted that our sample consisted of physically active adults, such as gym members and leisure exercisers, who are more likely to use dietary supplements than the general population. As a result, the prevalence may be influenced by self-selection bias, capturing a subset of the population that uses supplements more frequently. Nonetheless, psychological and sociocultural factors may also contribute (24, 25). Saudi adults, particularly youths, are increasingly characterized by health consciousness, motivation for self-enhancement, and investment in body image (26, 27), traits known to predict higher dietary supplement use (28, 29). Within self-determination and appearance-based motivation frameworks, dietary supplement use may reflect an effort to maintain perceived control over health and physical performance (30, 31). The growing influence of social media and the cultural emphasis on physical appearance likely reinforce these identity-driven behaviors (32).

### Age and anthropometric characteristics

Our findings indicated no significant differences in age, weight, height, or BMI between dietary supplement users and non-users, suggesting that the choice to take dietary supplements within the study population is primarily influenced by psychological or behavioral factors rather than physical attributes. This observation aligns with previous studies on recreationally active populations reporting no significant association between anthropometric indices and supplement use (5, 6, 33).

In our study, males exhibited significantly greater heights and weights compared to females (*η*^2^ = 0.20–0.47), consistent with recognized biological sex-based differences in body composition (16). However, sex-based differences in BMI were minimal (*η*^2^ = 0.02), indicating comparable body mass relative to height across sexes.

*Post-hoc* Tukey’s tests revealed small but significant differences in age and BMI across the sex × dietary supplement use groups. Regardless of their dietary supplement use status, females were generally younger than males (*p* = 0.003–0.005, *η*^2^ = 0.03), possibly reflecting a broader national trend of increasing fitness participation among younger women (34). Male users had marginally higher BMIs than both female users and non-users (*p* = 0.003–0.006), likely related to greater muscle mass or training intensity (33, 35). These minor differences did not correspond to higher dietary supplement use, reinforcing that body composition is not a primary driver of dietary supplement use.

Collectively, these results suggest that dietary supplement use among Saudi adults is more strongly associated with cognitive, motivational, and sociocultural factors than with physical characteristics, consistent with prior evidence emphasizing psychological predictors such as appearance orientation and self-enhancement (29, 36).

### Demographic characteristics

Most participants had a normal weight across all sex × dietary supplement use groups, whereas being underweight was more common among female users than among male users and non-users. This observation may indicate that sociocultural and psychological factors influence women’s perceptions of weight and their decisions to use dietary supplements. Research among young women in the region has found that body image concerns and exposure to social media are associated with weight-management behaviors, including dietary supplement use for aesthetic enhancement (29, 34, 37).

Significant associations were observed between dietary supplement use and body type, occupation, and education, but not monthly household income. Occupation exhibited the largest effect (*V* = 0.41), indicating that lifestyle and exposure to a gym culture play major roles. Higher education may indirectly influence supplement use by enhancing health awareness and facilitating access to occupations embedded in gym culture (38–40). Students and private-sector employees exhibited the highest use of dietary supplements, possibly due to peer influence and fitness engagement (16, 41). Conversely, those who were unemployed and government employees had lower use of dietary supplements, potentially reflecting differences in age, time availability, or routine PA. Although these interactions cannot be directly tested in the present cross-sectional analysis, they highlight potential mechanisms by which sociodemographic and lifestyle factors may shape supplement use, which will be explored further in Part II of our research project. The positive association between dietary supplement use and higher education aligns with studies showing that educated individuals exhibit greater health awareness and belief in the benefits of dietary supplements (17, 42). The lack of a significant effect for monthly household income suggests that lifestyle and knowledge, rather than financial capacity, are more influential determinants of dietary supplement use.

### Behavioral characteristics

Behavioral patterns, including training frequency and motivation, were generally similar between dietary supplement users and non-users but differed significantly by sex. Over half of the participants trained more than four times per week, reflecting high overall levels of PA in the sample. This observation aligns with national data showing rising exercise participation under Vision 2030 initiatives (34, 43).

Training frequency differed significantly across the sex × dietary supplement use groups [*χ*^2^ = 71.18, *p* < 0.001, *V* = 0.31 (medium effect size)]. Male users and non-users were more likely to train more than four times per week, whereas female non-users were more likely to exercise once a week or once a month. This pattern suggests that dietary supplement use is associated with greater training engagement, particularly among men, consistent with evidence linking dietary supplement use to training volume and gym attendance (33, 41, 44).

No significant difference in training motivation was observed across the sex × dietary supplement use groups (*χ*^2^ = 20.49, *p* = 0.154, *V* = 0.16). Most participants cited multiple motives for exercising, supporting the self-determination framework, which posits that intrinsic and extrinsic motivations frequently coexist (30, 31). Thus, dietary supplement use appears to correlate more with behavioral engagement, particularly training frequency, than with motivational orientation. The higher training frequency and dietary supplement use among males may reflect greater cultural and environmental access to sports facilities, while lower participation among females could stem from ongoing sociocultural barriers or time constraints (43). Overall, our findings emphasize the importance of sex-responsive health promotion to ensure equitable access to exercise and reliable information about dietary supplements.

### Limitations

Our study had several limitations that should be acknowledged. Firstly, its cross-sectional design prevents causal inference. Secondly, the self-reported data, including weight and height, may be affected by recall bias or social desirability, which could influence BMI calculations. Thirdly, the use of non-probability quota sampling limits the generalizability of our findings beyond the Eastern Province. Fourthly, nutrient intake or dietary supplement dosage was not assessed, which limits the interpretation of adequacy and safety. Notably, adverse effects and inadvertent doping will be discussed in our subsequent paper reporting Part II of the research project. Despite these limitations, the large and diverse sample examined in this study provides valuable insights into dietary supplement use patterns, supporting future longitudinal and interventional research.

## Conclusion

In our cross-sectional survey of physically active adults from the Eastern Province of Saudi Arabia, 66.6% reported using dietary supplements. Basic physical attributes (weight, height, and BMI) or most demographic variables did not differ significantly between users and non-users. Subgroup analyses revealed significant sex × supplement interactions in training frequency (*χ*^2^ = 71.18, *p* < 0.001, *V* = 0.31) and modest sex-specific differences in body type, occupation, and education (*V* = 0.12–0.41). *Post-hoc* Tukey’s tests indicated small but significant differences in age and BMI across sex × dietary supplement use groups (*η*^2^ ≈ 0.03). Therefore, our findings suggest that dietary supplement use is common and not primarily determined by anthropometric or income-related factors but is modestly shaped by sex-related training behaviors, occupational and educational contexts, and age-associated motivations.

Public health interventions should be tailored rather than uniform, focusing on high-frequency male exercisers and younger adults motivated by performance and aesthetics. Emphasizing label literacy, professional guidance, and regulatory oversight is crucial to ensuring safe and informed use of dietary supplements. Future research should explore psychological and informational factors, such as body-image concerns, health beliefs, and sources of advice, through longitudinal or mixed-method studies to better explain the widespread reliance on dietary supplementation despite minimal physical or socioeconomic differences.

## Data Availability

The raw data supporting the conclusions of this article will be made available by the authors, without undue reservation.
