# Supplementary material for: Patterns, motivations, and determinants of dietary supplement use among physically active adults in Eastern Saudi Arabia: a cross-sectional survey
Source: Front Public Health. 2026 Jan 22;14:1734477. doi: 10.3389/fpubh.2026.1734477 (PMC12872503; doi:10.3389/fpubh.2026.1734477)
Supplement: Supplementary file 2 [file Table_2.docx]

**Table S2**. Correlation between the current questionnaire and the Physical Activity Scale for Individuals with Physical Disabilities (PASIPD-AR).

| Questionnaire Components | Correlation Coefficient |
| --- | --- |
| Type of physical activity practiced | 0.25 |
| Purpose of supplement consumption | 0.18 |
| Adverse effects of supplement use | 0.12 |
| Frequency of supplement consumption | 0.14 |
| Attendance at physical activity and sports facilities | 0.21 |
| Perceived usefulness of supplements | 0.19 |
| Sources of supplement acquisition | 0.22 |
| Motivation for engaging in physical activities | 0.20 |
| Recommendation of supplements to others | 0.15 |
