# Supplementary material for: Patterns, motivations, and determinants of dietary supplement use among physically active adults in Eastern Saudi Arabia: a cross-sectional survey
Source: Front Public Health. 2026 Jan 22;14:1734477. doi: 10.3389/fpubh.2026.1734477 (PMC12872503; doi:10.3389/fpubh.2026.1734477)
Supplement: Supplementary file 3 [file Table_3.docx]

**Table S3**. Test–retest reliability coefficients of the questionnaire.

| Questionnaire Components | Correlation Coefficient |
| --- | --- |
| Overall reliability | 0.89 |
| Type of physical activity practiced | 0.970 |
| Purpose of supplement consumption | 0.978 |
| Adverse effects of supplement use | 0.993 |
| Frequency of supplement consumption | 0.964 |
| Attendance at physical activity and sports facilities | 0.822 |
| Perceived usefulness of supplements | 0.935 |
| Sources of supplement acquisition | 0.895 |
| Motivation for engaging in physical activities | 0.710 |
| Recommendation of supplements to others | 0.744 |
